# Colonization of Bone Flaps by Cutibacterium acnes During Decompressive Craniectomies for Traumatic and Non-traumatic Indications: A Retrospective Observational Study

**DOI:** 10.7759/cureus.71482

**Published:** 2024-10-14

**Authors:** Aishani Satia, Purushotham Ramanathan, Sebastian Salas-Vega, Sujata Ambardar, Mahesh Shenai

**Affiliations:** 1 Department of Neurosciences, Inova Health System, Falls Church, USA; 2 College of Medicine, University of Virginia, Charlottesville, USA; 3 Infectious Diseases Physicians, Inc., Inova Health System, Falls Church, USA

**Keywords:** bone flap infection, bone flap reimplantation, cutibacterium acnes colonization, decompressive craniectomy, postoperative care

## Abstract

Background

*Cutibacterium acnes* (*C. acnes*) is one of the most common bacteria in the human skin microbiota. Due to it generally requiring special culturing techniques, it was generally not routinely included in culture. However, recent laboratory automation advancements have allowed *C. acnes *to be a routinely tested pathogen. Though this could improve outcomes by detecting a virulent pathogen early, it has raised concerns about potential false positives leading to increased costs and medical risks. This study aims to analyze the *C. acnes *colonization rates in traumatic craniectomies and compare it to non-traumatic craniectomies, the former including risk factors due to penetrations and the latter being conceptually similar to a craniotomy with a lack thereof. This would help establish a baseline rate to understand the pathogen's implications better.

Methodology

We analyzed the electronic health records of 124 patients who underwent a craniectomy followed by a cranioplasty at Inova Health System from January 1, 2018, to January 1, 2023. The following categories of data were recorded for each patient: patient descriptors, comprehensive surgical timelines and outcomes, and bone flap viability and microbial colonization assessment. The chi-squared tests of independence and Wilcoxon signed-rank tests were used to assess statistical significance between groups in the indications underlying surgery (traumatic vs. non-traumatic) with *C. acnes* colonization, flap status (reimplanted vs. discarded), hospital length of stay (LOS), and unexpected 30-day readmission.

Results

Traumatic (67%) and non-traumatic (33%) craniectomies were compared. There was no significant association between the two craniectomy etiologies in terms of *C. acnes* colonization (40% vs. 26.5%, p=0.19), flap discardment (40% vs. 24%, p=0.12), or readmission rates (20% vs. 9.6%, p=0.18). However, a significant association was found between *C. acnes* colonization and LOS during the index craniectomy procedure (24.8 vs. 25.9 days, p=0.049), indicating that colonization may influence LOS. No significant association was found between the type of cranial surgery and LOS (p=0.83), suggesting other factors may play a more crucial role in determining LOS. The findings highlight the need to consider the impact of* C. acnes *colonization on surgical outcomes and hospital protocols.

Conclusion

Our findings illustrate that there is no significant difference between *C. acnes *colonization in traumatic and non-traumatic craniectomies; therefore, *C. acnes *can be expected to be cultured at a baseline level regardless of the etiology. Furthermore, there was no association with surgical indication and flap status, LOS, and readmission rates. However, a significant association was found between *C. acnes *status and LOS, indicating the increased complexity of care associated with the pathogen's detection. These findings support the protocol of deferring *C. acnes *culturing unless specific concerns are found.

## Introduction

*Cutibacterium acnes* (*C. acnes*) is a gram-positive bacterium and a well-known constituent of the human skin microbiota [1]. Despite its normal presence, in certain circumstances,* C. acnes* may produce virulent opportunistic infection [1]. As* C. acnes* generally requires special culture techniques and a delayed incubation time, it was generally not included in routine culture [2]. However, recently increased laboratory automation and proficiency have now included *C. acnes* as a routinely reported pathogen. While such detection may benefit the early detection of a virulent *C. acnes *infection, it is possible that overdetection may lead to false positives that will alter medical decision-making [3,4]. 

In neurosurgical practice, decompressive craniectomy (DC) is a commonly performed procedure, in which a portion of the skull is removed and stored for later re-insertion after resolution of a primary pathology, such as traumatic brain injury (TBI) or non-traumatic causes like middle cerebral artery (MCA) infarction or intracranial hemorrhage (ICH) [5]. After the removal of the skull, some institutions or surgeons will swab culture the bone flap, under the theory that positive cultures would preempt future reimplantation [6]. If this is the case, surgeons would opt to reconstruct the defect with either "mesh" or synthetic 3D printed cranioplasty. False-positive cultures would thus change the cranioplasty strategy [6]. 

At our institution, we noted an increasing number of *C. acnes* bone flap contaminations beginning in 2018, when our laboratory began routine testing for this microbe. This led to several isolated *C. acnes* detections, forcing a decision to discard the native bone flap. Such dilemmas can increase costs via the alternative of synthetic cranioplasty or impute medicolegal risk if a positive culture is dismissed. 

In order to understand the possibility of *C. acnes *testing leading to false positives, we wanted to understand the probability that any bone flap may be contaminated. In routine craniotomy (e.g., for tumors and aneurysms), bone flaps are not generally cultured and are expected to be immediately replaced at the time of surgery [7]. Thus, there is sparse culture data on these routine craniotomies. However, during non-penetrating or non-traumatic craniectomies (e.g., for stroke or intracranial hemorrhage), bone flaps were routinely cultured before storage, even though the inoculation risk to these bone flaps is no different than routine craniotomy. 

In the setting of penetrating trauma, there is a putative pathway for *C. acnes *and other microbes to directly inoculate the underlying bone flap. However, for non-traumatic or non-penetrating pathologies (e.g., stroke or intracranial hemorrhage), the method of bone flap removal is no different than a normal craniotomy, which does not require culture before bone flap replacement. As such, the microbial colonization of this non-traumatic subset of craniectomies is expected to mimic all craniotomies and provides a useful background upon which to compare traumatic craniectomies. 

This study aims to understand the presence and implications of *C. acnes* colonization in DC patients, differentiating between traumatic and non-traumatic craniectomy indications. A retrospective observational analysis was utilized to establish a baseline of *C. acnes *bone flap positivity rate from non-traumatic craniectomies and further compare that to bone flaps from traumatic craniectomies to assess whether traumatic incidents constitute a significant risk for *C. acnes* colonization. The conclusion that *C. acnes *is omnipresent could significantly alter hospital protocols for patient care by negating the necessity of discarding a viable native bone flap, shortening a patient's stay at the hospital, and saving medical expenses.

## Materials and methods

This investigation analyzed the electronic health records of 124 consecutive patients who underwent a DC followed by a cranioplasty at Inova Health System (Northern Virginia) within the study period from January 1, 2018, to January 1, 2023. This retrospective case series determined the sample size by conducting an electronic medical records search for all patients who met the inclusion criteria during the five-year period.

Approval was obtained from the Inova Health System Institutional Review Board (IRB) (approval number: U23-03-5030). The inclusion criteria for this study were as follows: craniectomy followed by cranioplasty, with the cranioplasty procedure occurring within the study period, both craniectomy and cranioplasty performed at Inova Health System, and availability of anaerobic cultures from the excised and preserved bone flap. The exclusion criteria for this study were as follows: bone flap excised but not preserved, unavailability of anaerobic bacteria cultures of the bone flap, craniectomy or cranioplasty procedures not performed at Inova Health System, patient mortality prior to cranioplasty, and cranioplasty procedures not occurring within the study period.

The following data was collected about each patient: patient identification details including medical record number, age at index craniectomy, and race; surgical details including date of craniectomy, date of cranioplasty, length of stay (LOS) for both procedures, surgical indication (traumatic vs. non-traumatic), and unexpected 30-day readmission of the patient; and bone flap status including *C. acnes* colonization and flap status (reimplanted vs. discarded). The electronic health records for each eligible patient were screened, and the relevant data was recorded in Excel (Microsoft Corporation, Redmond, Washington, United States). 

Statistical analyses were conducted using Python 3.10.0 (Python Software Foundation, Fredericksburg, Virginia, United States) in Google Colab. A chi-squared test of independence was performed to assess the association of the following relationships: indication (traumatic/non-traumatic) and *C. acnes* colonization, indication (traumatic/non-traumatic) and flap status (reimplanted/discarded), and indication (traumatic/non-traumatic) and unexpected 30-day readmission. The following data distributions were found to be non-normal distribution, and thus, a Wilcoxon signed-rank test was utilized to assess the following relationships: indication (traumatic vs. non-traumatic) and LOS during index cranioplasty and *C. acnes* colonization and LOS during index cranioplasty. A significance level of 0.05 was established.

At our institution, bone flaps are preserved according to a written policy, with the following key steps: Before freezing, bone flaps are soaked in a sterile solution of tobramycin and vancomycin and then aseptically packaged and labeled with accurate patient information. They are stored in an autologous bone freezer with continuous temperature monitoring and daily recordings. Initially, bone flaps are frozen at -20°C to -40°C for up to six months, after which the storage temperature is adjusted to -40°C to -89°C. Furthermore, according to institutional protocol, patients typically receive preoperative antibiotics, usually a second-generation cephalosporin, before the incision. Scalp preparation techniques generally include the application of ChloraPrep or DuraPrep for three minutes prior to draping, followed by Betadine solution. However, individual-level data on these variables were not systematically collected or analyzed in this study.

The methods employed in this study are designed to provide a comprehensive analysis of the factors influencing* C. acnes* colonization in DC. The primary analysis of interest is the rate of *C. acnes* colonization on the removed bone flaps in traumatic and non-traumatic cohorts. Furthermore, secondary analyses will illustrate how the etiology of the DC and colonization rates affect a patient's stay in the hospital, providing insight into the economic implications of post-surgical infections.

## Results

**Figure 1:**
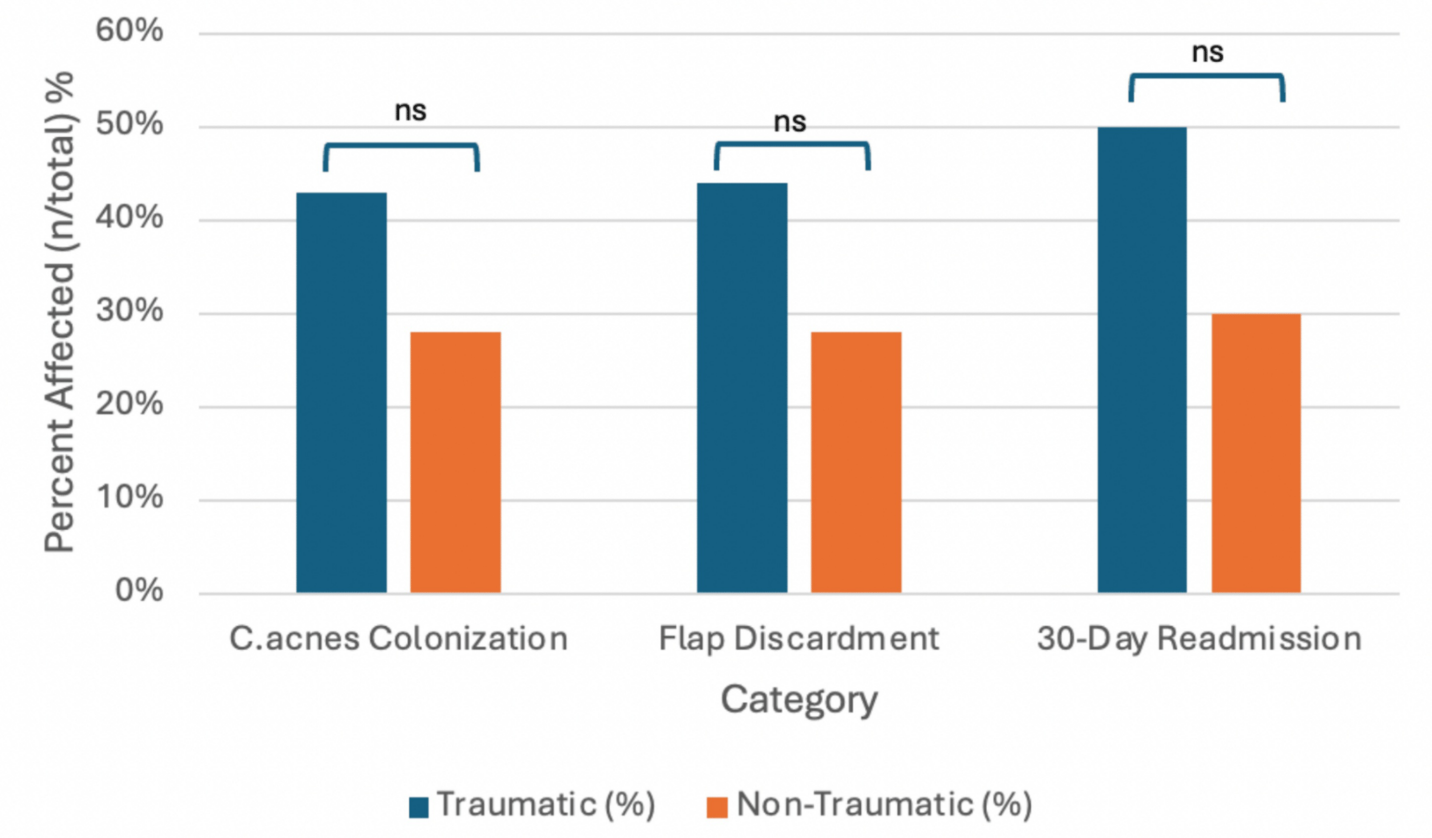
Percentage of patients affected by Cutibacterium acnes colonization, flap discardment, and 30-day readmission in traumatic versus non-traumatic craniectomies ns: non-significant

The demographic data including average index age, race, and gender distribution between the traumatic and non-traumatic craniectomy cohorts is summarized in Table 1. The analysis revealed no statistically significant associations between the type of cranial surgery indication (traumatic or non-traumatic) and *C. acnes* colonization, flap status (reimplantation or discard), or unexpected 30-day readmission (Figure 1, Table 2). However, a significant association (p=0.05) was identified between the surgical indication and LOS during the index cranioplasty procedure (Table 3). This suggests that patients with *C. acnes* colonization may have a different LOS than those without. Interestingly, no significant association was found between the type of cranial surgery indication (traumatic or non-traumatic) and LOS (tested with Wilcoxon signed-rank test, p=0.083). This finding suggests that factors other than the initial reason for surgery may be more influential in determining LOS. 

**Table 1:** Demographic and clinical characteristics of patients undergoing traumatic vs. non-traumatic craniectomies

	Traumatic craniectomies, n=83 (67%)	Non-traumatic craniectomies, n=40 (33%)
Average index age	52	36
Race (non-White) (N, %)	46 (55%)	22 (55.4%)
Gender (% female) (N, %)	9 (22.5%)	43 (51.8%)

**Table 2:** Clinical outcomes and associated p-values for traumatic and non-traumatic craniectomies

	Traumatic craniectomies, n=83 (67%)	Non-traumatic craniectomies, n=40 (33%)	P-value	Chi value	Wilcoxon test statistic
*Cutibacterium acnes *colonization (N, %) (95% CI)	33 (40%) (34-46%)	11 (26.5%) (15.2-37.8%)	0.19	1.71	N/A
Flap infection (N, %) (any cause) (95% CI)	39 (47.5%) (41.3-53.7%)	11 (27.7%) (16.3-39.1%)	0.05	3.86	N/A
Flap status (N, % discarded) (95% CI)	33 (40%) (34-46%)	10 (24%) (13.1-34.9%)	0.12	2.44	N/A
Unexpected 30-day readmission (N, % readmitted) (95% CI)	17 (20%) (15.1-24.9%)	4 (9.6%) (2.1-17.1%)	0.18	1.81	N/A
Average length of stay (days) (95% CI)	28.5 (18.6-38.4%)	24.1 (21.2-27%)	0.83	N/A	372.5

**Table 3:** Association between Cutibacterium acnes colonization and length of stay

	*Cutibacterium acnes *colonization present	*Cutibacterium acnes *colonization absent	P-value	Wilcoxon test statistic
Average length of stay (days) (95% CI)	24.8 (19.6-30%)	25.9 (20.9-30.9%)	0.049	208.5

## Discussion

This study aimed to understand the relationship between indication and the risk of colonization of *C. acnes* in native bone flaps following a DC. The results support the notion that there is no significant difference in *C. acnes* colonization between traumatic and non-traumatic indications for DC. While the former may have infectious risk factors due to penetrating injuries and multisystem trauma, the latter should be conceptually similar to general craniotomy by lack of those risk factors. As there is no significant difference between the traumatic and non-traumatic presence of *C. acnes*, by inference, *C. acnes *can be expected to be cultured on any bone flap at a baseline level, regardless of indication. Thus, the practice of discarding native bone flaps, specifically due to the presence of *C. acnes* alone, should, therefore, be challenged. 

During routine craniotomy, such as for tumor or vascular indications, it is impractical to test the bone flap for infection, and thus, it is immediately replanted during the same surgery. While further studies are required to quantify and confirm the colonization rate on these types of craniotomies, we expect it to be identical to the rate found on non-traumatic craniectomies. By the same logic, native bone flaps from craniectomy without any evidence of gross contamination should be replanted, without the need for routine culture. However, if there is evidence of gross contamination from a penetrating injury or a severe structural defect of the flap, such as a comminuted fracture, then it should be discarded, but not on the basis of one culture [8]. Multiple studies have demonstrated that culture alone is not a reliable indicator of true infection and detection methods for *C. acnes* are often inaccurate [9-12]. This is particularly relevant as *C. acnes* is a common bacterial contaminant on bone flaps [13]. Khalil et al. attributed discrepancies in culturing results to changes in diagnostic procedures and extended incubation times [14]. 

Conversely, a study by El Soufi et al. reported a rare case of *C. acnes *complicating a subdural hematoma evacuation, concluding that the presence of this bacterium, although rare, signifies a true infection [15]. The study further highlights the dual nature of *C. acnes* detection, its typically non-pathogenic nature, and the challenges in distinguishing between true infection and gross contamination. They similarly recommend maintaining a high index of clinical suspicion and suggest obtaining multiple specimens from the site.

With regard to secondary outcomes, there was no association with surgical indication and flap status, LOS, and readmission rates. However, the presence of *C. acnes *was associated with a greater LOS. Consequently, it can be expected that detection of *C. acnes* will be associated with a higher cost of care, due to the LOS and the complexity of care, in addition to the extra material cost of a synthetic skull prosthetic. In correlation, Hoang et al. observed that when culture results are not accurately interpreted, it can lead to unnecessary antibiotic treatments and a higher incidence of synthetic bone flaps [16].

At our institution, we followed a historical policy in which any microbial presence on the bone flap culture mandated discarding the flap in favor of synthetic cranioplasty. As *C. acnes* detection became available and produced more positive cultures, we recently revisited this policy out of concern for its futility and increased cost of care. Currently, we do not perform a mandatory culture of harvested bone flaps during the index procedure, unless requested by the surgeon for unique circumstances. This is in line with a recent meta-analysis, which found no difference in infection rates based on native versus synthetic cranioplasty [17,18]. 

Limitations of this study include the relatively small sample size and the retrospective and observational nature of the study, which could introduce bias. For example, traumatic and non-traumatic craniectomy patient populations may differ significantly in age, gender, and other factors. While we did not detect a significant difference in either our primary or secondary outcomes, one may exist in a clinically relevant subset. Specific patient-related comorbidities, such as diabetes mellitus, could also further impact the risk of infection and induce distinct rates of wound healing. Additional research could stratify the data into subsets based on these characteristics. Notably, traumatic craniectomies were not classified based on the presence or extent of penetrating or comorbid injuries, which may be a subset of patients that require special concern. Future studies would be required to determine if a policy of non-testing has any impact on eventual infection, readmission, LOS, or mortality. Potential confounders in this study include the lack of consistent documentation of open scalp injuries overlying the skull flap site, variability in the use of antimicrobials, and the type of disinfectants used during scalp preparation on an individual basis. Addressing these limitations would help provide more robust insights and further insights into the bacterium's impact in a neurosurgical setting.

## Conclusions

This study provides valuable insights into the prevalence and implications of *C. acnes* colonization on the bone flap post-DC. The lack of a significant association between craniectomy indications and *C. acnes* colonization suggests that *C. acnes* may be present in all craniotomies, which nevertheless get reimplanted. These findings support a reasonable policy of deferring mandatory bone flap culturing unless there is gross contamination or other specific concerns. Additional studies are needed to assess the effectiveness of bone flap culturing policies.
